# The impacts of diagnosis-intervention packet payment on inpatient medical costs for hematologic malignancies and solid tumors: evidence from a retrospective study in China

**DOI:** 10.3389/fpubh.2025.1453367

**Published:** 2025-05-07

**Authors:** Huiping Xu, Qunqing She, Beibei Zhang, Shaogui Zhang, Linjun Xie

**Affiliations:** ^1^The First Hospital of Putian City, Putian, China; ^2^The School of Clinical Medicine, Fujian Medical University, Fuzhou, China

**Keywords:** diagnosis-intervention packet, hematologic malignancies, solid tumors, inpatient medical costs, China

## Abstract

**Background:**

This study aims to analyze and compare the impact of the diagnosis-intervention packet (DIP) payment on inpatient medical costs for hematologic malignancies (HM) and solid tumors (ST) patients, and to explore its implications for hospital financial sustainability and payment reform.

**Methods:**

Using a retrospective research design, this study focused on HM and ST patients treated before and after the implementation of the DIP payment at a large tertiary general hospital in A city, located in the eastern coastal area of China. Data were collected, organized, and analyzed to compare differences in inpatient medical costs between HM and ST patients and to examine their impact on the income of the department of hematology.

**Results:**

The study included 5,115 cases from both before and after the DIP payment implementation. Post-implementation, the median inpatient medical costs per case decreased from 5,544.45 CNY to 5,169.66 CNY, with costs for both HM and ST hospitalizations showing a decline. Specifically, the inpatient medical costs per case for HM were 5,722.46 (4,471.08, 11,508.78) CNY, higher than those for ST at 4,779.28 (3,056.70, 7,152.64) CNY, and exceeding the DIP payment standard. Wilcoxon signed-rank test and regression analysis indicated that HM inpatient medical costs surpass the standard payments, resulting in financial losses. All findings were statistically significant (*p* < 0.05). These results suggest a structural mismatch between DIP reimbursement rates and the resource intensity of hematologic malignancy treatment, which may jeopardize the financial viability of hematology departments.

**Conclusion:**

Despite a reduction in median inpatient medical costs following the implementation of the DIP payment, departments treating HM patients continue to experience financial losses due to costs exceeding the payment standard. These findings highlight the need to refine DIP payment standards to better account for clinical complexity and technological advancements. Future reforms should aim to improve alignment between payments and actual care needs to ensure financial sustainability and equity. However, this study is limited by its single-center design and lack of control for potential confounders. Broader multi-center studies with more detailed clinical data are needed to validate and extend these findings.

## 1 Introduction

In recent years, as healthcare costs have risen and resource allocation has become more complex, diagnosis-related group (DRG) payment systems have gained increasing attention and widespread use globally (1). Initially proposed in the United States and subsequently implemented in many European and American countries, DRG systems have demonstrated significant effectiveness in controlling hospital costs, optimizing health insurance payments, and improving the efficiency of healthcare services (2, 3). To curb the rapid growth of healthcare expenditures, China introduced the diagnosis-intervention packet (DIP) payment system in 2020 (4). The DIP payment system, while similar to DRG system, distinguishes itself by emphasizing the integration of primary diagnoses and treatment methods through big data analysis. By establishing case combinations and payment standards, a unified standard system and resource allocation model were created to enhance the efficiency of health insurance fund utilization, rationally guide the allocation of healthcare resources, and ensure the basic medical needs of insured individuals. This innovative case-based payment method aims to replace the traditional fee-for-service model and address issues related to the DRG system’s coverage and demand behavior (5, 6).

After the implementation of the DIP payment system in China, research has shown that it effectively curbed the growth of healthcare expenditures, promoted the rational allocation of healthcare resources, and reduced unreasonable practices such as overtreatment and induced demand (7–9). However, the majority of these studies have concentrated on the system’s overall economic impact and operational feasibility, with limited attention paid to patient subgroups with rare or high-cost diseases. For instance, hematologic malignancies (HM) are a minority within the category of malignant tumors and are grouped together with the entire oncology population under the DIP payment system. The policy sets a standardized payment amount for patients with previously diagnosed malignant tumors undergoing maintenance chemotherapy, without differentiating between HM and solid tumors (ST) despite significant disparities in their pathophysiology, treatment regimens, and inpatient medical costs (10, 11). This raises important concerns about fairness in health financing under the DIP payment system. The existing literature has not adequately explored whether the “averaging effect” inherent in big-data-driven payment systems may unintentionally disadvantage vulnerable populations such as HM patients.

In this context, this study selects a large tertiary general hospital (referred to as Hospital A) in A City, a pilot city for DIP payment reform in the eastern coastal area of China, as a case study. The aim is to investigate the trends and differences in inpatient medical costs for HM and ST patients under the DIP payment system and to analyze its impact on the revenue of the department of hematology. The findings intend to inform more equitable payment reforms that better align reimbursement standards with clinical realities and patient needs.

## 2 Methods

### 2.1 Study design

This study adopted a retrospective design based on inpatient data from Hospital A. Hospital A is a Class A tertiary hospital with over 1,500 beds and comprehensive clinical departments, including a well-established hematology unit with a high volume of HM cases. As one of the early pilot institutions for the DIP payment reform, Hospital A was selected due to its policy representativeness and the availability of complete and continuous DIP payment data both before and after the policy implementation, which allowed for a longitudinal assessment of its impact. Leveraging this dataset, the study focused on comparing inpatient medical costs between patients with HM and those with ST under the DIP payment system, and further explored the financial implications for the hematology department.

### 2.2 Data sources

This study utilized hospital admission records from Hospital A for HM and ST patients treated between 2021 and 2023, encompassing patient characteristics, hospitalization duration, diagnoses, and inpatient costs. 2022 marked the DIP implementation transition; 2021 and 2023 data served as pre- and post-implementation samples, respectively (note: some HM cases originated from departments outside hematology). The study also analyzed 2023 revenue details of Hospital A’s hematology department under the DIP payment system.

### 2.3 Sample selection

Inclusion criteria for this study were as follows: (1) Patients diagnosed with HM or ST, with HM types including acute leukemia (AL), lymphoma, and multiple myeloma (MM); (2) Primary diagnosis on the first page of the medical record indicating maintenance chemotherapy for malignant tumors (Z51.103), tumor chemotherapy course (Z51.100), malignant tumor immunotherapy (Z51.800), or targeted therapy for malignant tumors (Z51.801); (3) Availability of complete clinical records and treatment documentation. Exclusion criteria included: (1) Discharge within 24 h without complete clinical data; (2) Presence of severe chronic diseases or complications resulting in hospital stays exceeding 45 days; (3) Single hospitalization costs exceeding CNY 180,000; (4) Cases requiring intensive care or multidisciplinary treatment.

### 2.4 Statistical analysis

Data processing utilized Python version 3.12 with pandas and SciPy libraries. Categorical data were summarized with counts and percentages, analyzed using chi-square tests. Normally distributed continuous data were presented as mean ± standard deviation and compared using independent or paired t-tests. Non-normally distributed continuous data were expressed as median (Q1, Q3) and analyzed with Mann–Whitney U or Wilcoxon signed-rank tests. Matplotlib and seaborn libraries facilitated data visualization. Statistical significance was defined as *p* < 0.05.

### 2.5 Ethical considerations

Patient privacy and confidentiality were paramount throughout this study. All patient information was de-identified and securely managed. Access to data was restricted to authorized personnel only, and electronic medical records were stored on a password-protected secure server. Data analysis was conducted in aggregate form to prevent the identification of individual patients.

## 3 Results

### 3.1 Overall trends in hospitalization days and costs pre- and post-DIP payment implementation

This study included 2,247 cases before and 2,868 cases after the implementation of DIP payment in the city A. There were no significant differences in gender and age between the two groups, with a median hospitalization duration of 5 days for both groups. The median inpatient medical costs per case decreased from 5,544.45 (3,449.39, 8,960.18) CNY in 2021 to 5,169.66 (3,184.57, 7,657.74) CNY in 2023, representing a statistically significant 6.76% reduction (*p* < 0.001). Regarding the cost structure, medical service and consumables costs increased, while treatment, western medicine, and other costs decreased (*p* < 0.001). Detailed breakdown and changes in cost structure for both groups are shown in Table 1.

**Table 1:** Trend analysis of inpatient medical costs per case and structural changes pre- and post-DIP payment implementation (costs in CNY).

Characteristics	Pre-DIP payment (2021)	Post-DIP payment (2023)	Difference	*p*
Female (%)	1,018 (45.30%)	1,311 (45.71%)	–	0.794
Age (years)	62.30 ± 11.38	62.53 ± 11.19	–	0.455
Hospitalization days	5.00 (3.00, 8.00)	5.00 (2.00, 8.00)	–	–
Medical service costs	340.00 (166.75, 525.50)	447.50 (199.38, 865.08)	31.62%	<0.001
Diagnostic costs	893.00 (549.50, 1800.50)	900.75 (508.00, 1840.38)	0.87%	0.366
Treatment costs	186.10 (89.20, 320.35)	0.00 (0.00, 123.78)	−100.00%	<0.001
Western medicine costs	3082.57 (1504.26, 6189.44)	2591.12 (1218.99, 5176.36)	−15.94%	<0.001
Traditional Chinese medicine costs	0.00 (0.00, 55.50)	0.00 (0.00, 0.00)	–	–
Blood products costs	0.00 (0.00, 0.00)	0.00 (0.00, 0.00)	–	–
Consumables costs	40.76 (0.00, 260.66)	68.12 (29.01, 123.14)	67.14%	<0.001
Other costs	126.00 (72.00, 227.13)	0.00 (0.00, 72.00)	−100.00%	<0.001
Inpatient medical costs (Total costs)	5544.45 (3449.39, 8960.18)	5169.66 (3184.57, 7657.74)	−6.76%	<0.001

### 3.2 Subgroup comparison between HM and ST groups

**Figure 1:**
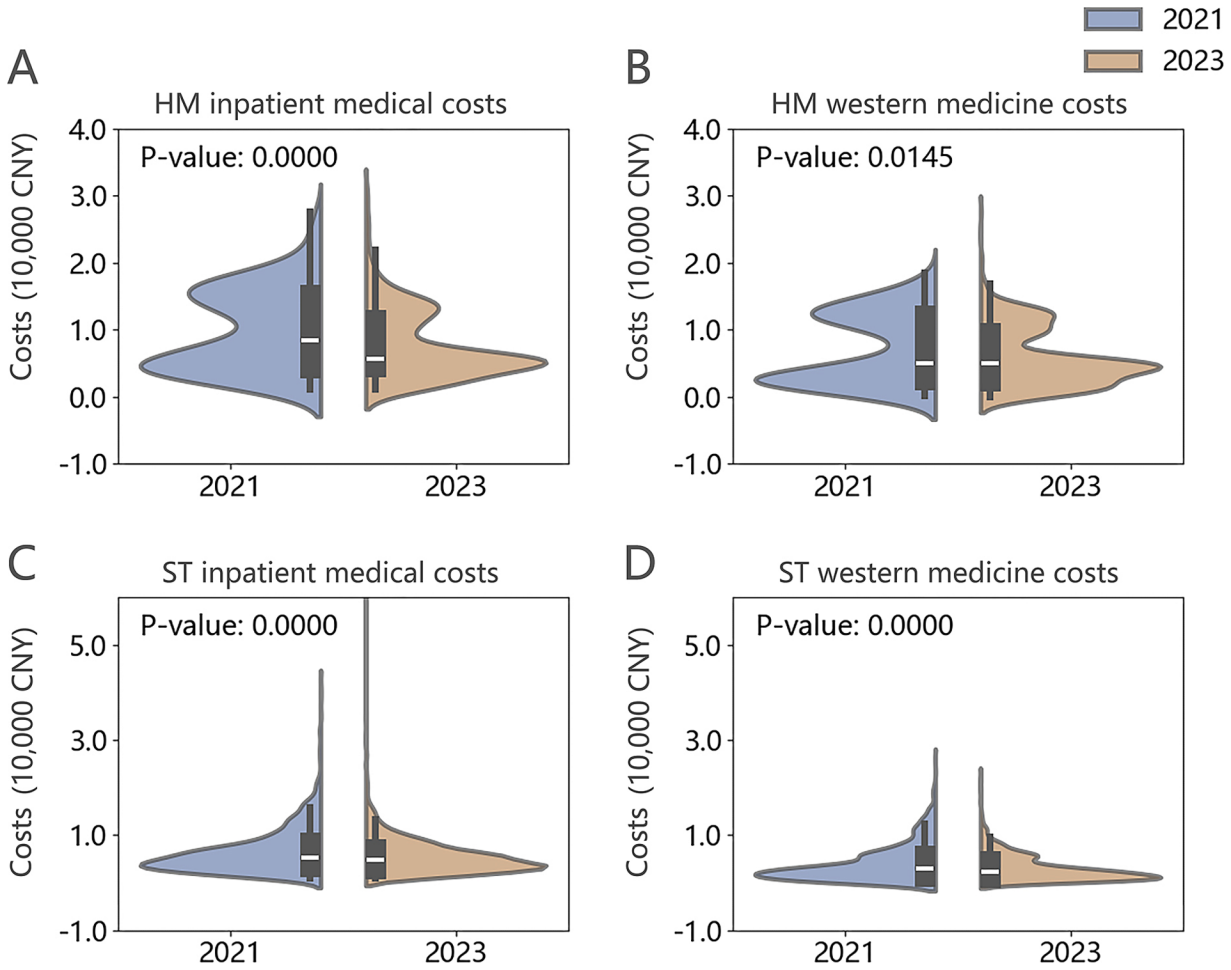
**(A–D)** Violin plots of inpatient medical and western medicine costs per case for HM and ST groups in 2021 and 2023.

The two groups of patients were divided into HM and ST groups, with 246 cases in the HM group and 2,001 cases in the ST group before the implementation of DIP payment. After implementation, there were 606 cases in the HM group and 2,262 cases in the ST group. Implementation of DIP payment led to statistically significant reductions in both inpatient medical and western medicine costs for both the HM and ST groups compared to pre-implementation levels (*p* < 0.05). Additionally, it can be observed that the cost distribution in the HM group exhibits a distinct bimodal pattern compared to the ST group (Figures 1A–D).

Before and after the implementation of the DIP payment system, there were no significant differences in gender and age characteristics between HM and ST patients. In 2021, patients in the HM group had a median hospitalization duration of 7 days and median inpatient medical costs of 8,459.62 CNY, both significantly higher than those in the ST group (*p* < 0.001). The median western medicine cost per case for HM patients was also notably higher, at 4,951.32 CNY (*p* < 0.001). By 2023, the median hospitalization duration for the HM group had decreased to 3 days, shorter than that of the ST group. Median inpatient medical costs per case declined to 5,722.46 CNY, while western medicine costs slightly increased to 4,966.32 CNY. Despite the overall reductions in hospitalization duration and total inpatient costs, western medicine costs for HM patients remained significantly higher than those in the ST group (*p* < 0.001). Detailed data are presented in Table 2.

**Table 2:** Comparison of hospitalization days and inpatient medical costs per case between HM and ST groups pre- and post-DIP payment implementation (costs in CNY).

	Pre-DIP payment (2021)			Post-DIP payment (2023)		
	HM	ST	*p*	HM	ST	*p*
Female (%)	105 (42.68%)	913 (45.63%)	0.419	285 (47.03%)	1,026 (45.36%)	0.492
Age (years)	61.28 ± 13.27	62.42 ± 11.12	0.136	63.06 ± 11.62	62.39 ± 11.07	0.191
Hospitalization days	7.00 (4.00, 9.00)	5.00 (3.00, 8.00)	<0.001	3.00 (1.00, 7.00)	5.00 (2.00, 8.00)	<0.001
Inpatient medical costs	8459.62 (4291.66, 15211.18)	5379.33 (3350.56, 8379.23)	<0.001	5722.46 (4471.08, 11508.78)	4779.28 (3056.70, 7152.64)	<0.001
Western medicine costs	4951.32 (2515.50, 12091.61)	2899.93 (1403.14, 5836.70)	<0.001	4966.32 (2383.89, 9490.56)	2278.44 (1044.07, 4496.58)	<0.001

### 3.3 Subgroup comparison within the HM group

Before and after the implementation of the DIP payment system, both the inpatient medical costs and western medicine costs per case for the HM group decreased, with Mann–Whitney U test analysis indicating statistically significant differences. Analysis of case composition within the HM group in 2023 revealed a notable increase in MM cases, accompanied by a rise in both median inpatient medical and western medicine costs. However, these differences were not statistically significant. Conversely, lymphoma cases showed a decrease in both inpatient medical and western medicine costs (*p* < 0.05). Detailed data can be found in Table 3.

**Table 3:** General information of cases in HM group (costs in CNY).

		Pre-DIP payment (2021)	Post-DIP payment (2023)	*p*
AL	Cases	39 (15.85%)	46 (7.59%)	–
	Inpatient medical costs	6034.18 (3251.88, 8154.86)	6015.36 (4876.62, 8164.05)	0.182
	Western medicine costs	4164.89 (1967.29, 4994.85)	4007.58 (2893.49, 5188.91)	0.390
Lymphoma	Cases	170 (69.11%)	220 (36.30%)	–
	Inpatient medical costs	13264.41 (5949.89, 16462.09)	11319.76 (5974.81, 13834.43)	0.002
	Western medicine costs	11146.55 (2657.63, 13105.66)	9632.50 (3844.62, 12003.49)	0.046
MM	Cases	37 (15.04%)	340 (56.11%)	–
	Inpatient medical costs	4016.11 (3430.16, 6281.97)	5215.74 (3012.97, 5957.96)	0.107
	Western medicine costs	3217.52 (2343.36, 4553.01)	4201.37 (1609.64, 5154.28)	0.061
Total	Inpatient medical costs	8459.62 (4291.66, 15211.18)	5722.46 (4471.08, 11508.78)	<0.001
	Western medicine costs	4951.32 (2515.50, 12091.61)	4966.32 (2383.89, 9490.56)	0.015

### 3.4 Analysis of the relationship between costs, hospitalization days, and age in the HM group, and intra-group differences

**Figure 2:**
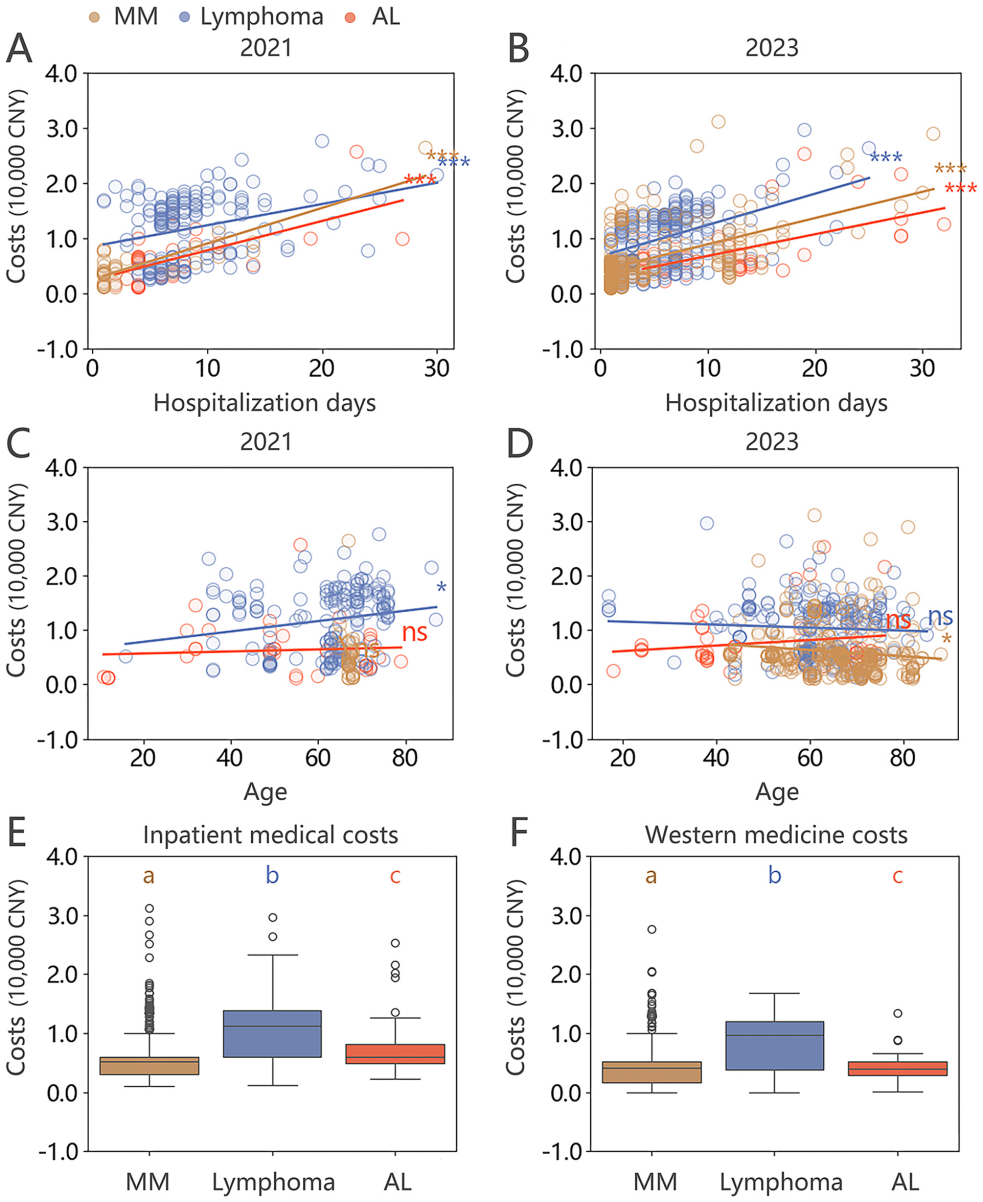
**(A–D)** Scatter plots and fitted lines for hospitalization days, age, and inpatient medical costs per case in the HM group for 2021 and 2023 (Mann–Whitney U test: ns no statistical significance; * *p* < 0.05; ** *p* < 0.01; *** *p* < 0.001). **(E,F)** Box plots of inpatient medical costs and western medicine costs for different types of HM in 2023, with Mann–Whitney U test indicating significant differences between each subgroup (denoted by abc, *p* < 0.05).

Analysis of scatter plots and fitted lines for inpatient medical and western medicine costs within the HM group in 2021 and 2023 (Figures 2A–D) revealed a positive correlation with hospitalization days, although the slopes differed between years. Notably, there was an increase in MM cases in 2023, concentrated among individuals aged 40–60. While no overall age trend was observed, specifically, lymphoma costs in 2021 showed a positive correlation with age, while MM costs in 2023 exhibited a negative correlation with age (Figures 2C,D, *p* < 0.05). Further analysis within the HM group demonstrated statistically significant differences in inpatient medical and western medicine costs across different subtypes of HM (Figures 2E,F).

### 3.5 Analysis of the 2023 financial balance in the department of hematology under the DIP payment system

**Figure 3:**
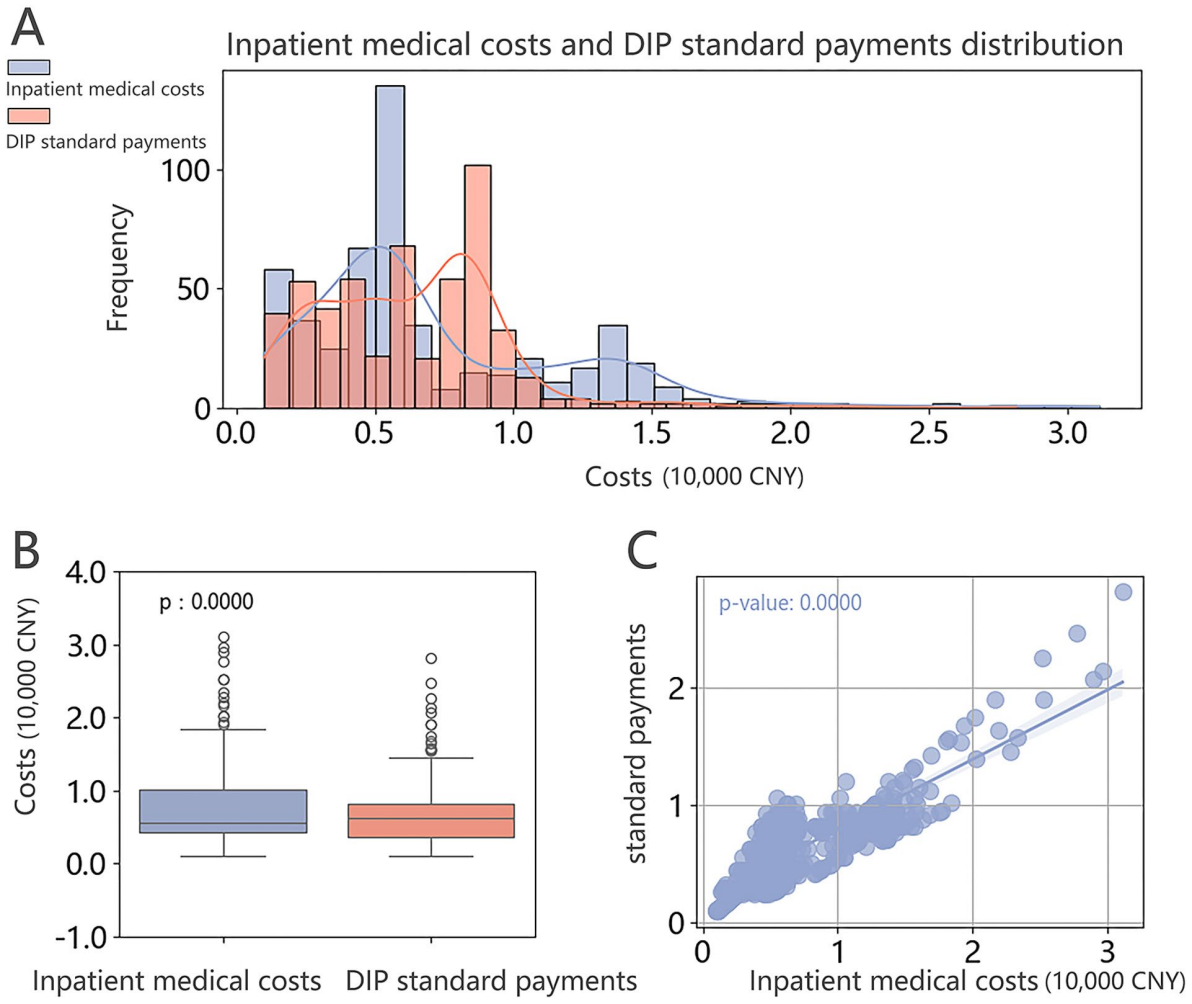
Comparison of inpatient medical costs and DIP standard payments per case in the HM group. **(A)** Distribution plot; **(B)** Box plot showing the median and interquartile range, with the Wilcoxon signed-rank test indicating statistically significant differences between groups. **(C)** Scatter plot with fitted line showing the trend.

In 2023, a total of 1,429 cases from the Department of Hematology were included in the analysis, with total inpatient medical costs reaching 8,935,167.84 CNY and corresponding DIP standard payments amounting to 8,398,670.79 CNY. This resulted in an overall departmental deficit of 536,497.05 CNY, among which 529 cases were attributed to the HM group (Figure 3A). For the HM group, total inpatient medical costs were 3,827,303.52 CNY, while DIP standard payments amounted to 3,363,359.22 CNY, leading to a deficit of 463,944.30 CNY—accounting for 86.48% of the department’s total loss.

At the case level, the median inpatient medical cost per HM case was 5,540.46 CNY (4,350.89, 10,198.37), compared to a median DIP standard payment of 6,300.00 CNY (3,560.37, 8,250.00). Although the median standard payment was marginally higher, the wider interquartile range suggests that actual inpatient costs often exceeded reimbursement. The Shapiro–Wilk test indicated a non-normal distribution of cost differences, which was further supported by a statistically significant result from the Wilcoxon signed-rank test (Figure 3B).

A scatter plot illustrating inpatient medical costs versus standard payments revealed a negatively sloped fitted line, suggesting that higher inpatient expenditures were associated with greater financial deficits (Figure 3C, *p* < 0.05). Notably, the bimodal distribution observed in the HM group’s cost structure may be a contributing factor to this imbalance.

## 4 Discussion

President Xi Jinping has pointed out, “The principal contradiction in Chinese society has evolved into the contradiction between the people’s ever-growing needs for a better life and unbalanced and inadequate development (12).” This perspective is also reflected in medical diagnosis and treatment. The increasing demand for novel and efficient treatment methods by patients has resulted in a significant gap between the allocation of medical resources and the pace of technological advancements (13, 14). In “Contradiction Theory,” Comrade Mao Zedong emphasized that contradictions are ubiquitous and serve as the fundamental driving force for development. In recent years, China has significantly increased healthcare expenditure, expanded healthcare coverage, and established special funds to pay for high-value drugs and innovative medicines (15). Additionally, national centralized drug procurement and price negotiations have substantially reduced drug prices, gradually alleviating the financial burden on patients during treatment (16). Moreover, DIP payments have leveraged the characteristics of China’s medical resources. Through big data analysis and intelligent algorithms, DIP payments achieve precise classification of diseases and optimized allocation of resources, showing great potential in promoting the rational use of medical resources and enhancing service levels (17).

City A was chosen as a pilot city for the total budget model, utilizing regional point methods and DIP payments, beginning trial implementation in 2022. Our research demonstrates that after the implementation of the policy, both the inpatient medical and western medicine costs at Hospital A have decreased, while medical service fees have increased, consistent with reports from other studies in China (7, 8). Zhang et al. (18) also examined the impact of the DIP payment policy on hospitalization costs for cancer patients, reporting a significant reduction in total inpatient expenses, particularly in drug-related costs. At the same time, expenditures on technical medical services increased, suggesting a gradual shift toward value-based healthcare. These findings align with our results, indicating that the DIP model may effectively curb irrational drug use and incentivize hospitals to reallocate resources toward professional service provision. Similarly, Liu et al. (19) found that the DRG-based payment reform in China contributed to shorter average lengths of stay and lower hospitalization costs, demonstrating comparable cost-containment outcomes under different payment models. However, the emergence of similar results across diverse regions and reform types raises concerns about potential unintended consequences, such as cost-shifting behaviors or selective admission practices (20, 21). This highlights the importance of enhancing the dynamic adjustment of point-value standards and reinforcing supervision mechanisms to ensure that payment reforms lead to sustainable and equitable improvements in healthcare delivery.

Unlike most existing studies that primarily assess the overall impact of DIP reform at the hospital or regional level, this study innovatively focuses on disease-specific cost disparities, particularly between HM and ST patients, which have received limited attention in prior research. Our analysis found that while inpatient medical and Western medicine costs for the HM group have decreased compared to previous periods, they remain significantly higher than those for the ST group. The treatment methods for ST are relatively mature, leading to more stable cost growth, whereas the diagnosis and treatment of HM have progressed rapidly (11). The swift advancement in targeted and immunotherapeutic strategies has markedly augmented personalized treatments for HM, leading to impressive improvements. Innovations such as CAR-T cell therapy and cutting-edge targeted medications, including Bruton’s tyrosine kinase inhibitors, Bcl-2 inhibitors, and CD38 monoclonal antibodies, have the potential to significantly boost survival rates and the quality of life for patients (22–25). Nonetheless, these pioneering treatments tend to be costly, precipitating a sharp increase in the expenses associated with HM treatment. It is noteworthy that, despite a reduction in expenses for other cancer types such as AL and lymphoma, there has been a notable escalation in the number of MM instances, along with the median costs. This trend could be attributed to an increased prevalence of MM, updates in treatment regimens, and the introduction of new pharmaceuticals (26, 27). While the advent of these novel therapeutic options and drugs enhances treatment efficacy, it concurrently escalates the treatment expenditure.

The DIP payment system utilizes a total budget model, assigning a point value to each medical institution based on annual medical insurance payments, insurance payment ratios, and overall case scores. This system establishes standardized payments per case. Currently, this policy determines scores and makes DIP standard payments for the maintenance treatment of various types of malignant tumors based solely on different treatment methods. However, payment standards based solely on historical costs may not adequately account for the rapid advancements in HM treatments, potentially resulting in underpayments (28). Our study corroborated this concern. In 2023, after the implementation of the DIP payment system, the hematology department of Hospital A experienced a loss of 463,944.3 yuan in the HM group, accounting for 86.48% of the total loss. If this situation persists, the actual costs of medical services cannot be effectively compensated, which may demotivate frontline medical staff and affect the quality of medical services, as well as the sustainable development of the deficit department. Ensuring fair reimbursement for these two types of tumors within the DIP payment framework has become a pressing issue that needs to be addressed.

Contradictions exhibit both universality and particularity. The unique clinical and economic challenges associated with the treatment of HM, in contrast to ST, are clearly reflected in the substantial financial losses faced by hematology departments under the current DIP system. To address this specific issue, it is essential to adjust medical insurance payment standards accordingly. In this regard, international DRG-based payment systems offer valuable lessons. For example, Germany’s G-DRG system incorporates patient clinical complexity levels that account for comorbidities and complications, allowing for differentiated reimbursement even within the same diagnosis group (29). For advanced therapies like CAR-T and allo-HSCT, DRG-based reimbursement varies considerably, and the use of comorbidity indices such as the Charlson Comorbidity Index improves the assessment of clinical complexity, length of stay, and total treatment costs (30). These mature systems demonstrate that incorporating severity and treatment complexity indicators is critical to achieving both payment fairness and efficiency. In contrast, China’s DIP system still lacks sufficient risk adjustment mechanisms, which may result in undercompensation for resource-intensive subgroups like HM patients.

Therefore, future reforms should focus on refining DIP grouping and scoring rules by integrating indicators of clinical severity, treatment intensity, drug specificity, and supplementary scores based on tumor characteristics (e.g., secondary diagnoses). This would improve the alignment between reimbursement levels and actual resource consumption. Such reforms would help capture evolving clinical practices more accurately, promote rational resource allocation, and better support the delivery of high-quality care.

This study relied solely on data from a single tertiary hospital due to limitations in data access. This single-center design presents several challenges, including limited sample representativeness, a restricted study period, potential concerns about data integrity and quality, and an insufficient exploration of diverse treatment approaches. In addition, the study lacked control for potential confounding factors, such as variations in disease severity, treatment regimens, or patient socioeconomic status, which may have influenced cost outcomes. These limitations may affect the generalizability and reliability of the findings. Future research should address these issues by incorporating data from multiple centers, extending the study duration, applying multivariable analytical approaches to control for confounders, and enhancing data quality. Such improvements will provide a more robust foundation for evidence-based medical decision-making.

## 5 Conclusion

This study offers a comprehensive analysis of the effects of DIP payments on inpatient medical costs across different tumor types and the financial outcomes within the hematology department at Hospital A. Our findings reveal that although DIP payments have successfully lowered the overall inpatient medical costs and expenses related to western medicine, the treatment expenses for the HM group remain substantially higher compared to those for the ST group, resulting in financial losses for the department. The study highlights the financial losses incurred by minority groups due to the averaging effect of big data, emphasizing the need for policy adjustments. Future initiatives should concentrate on refining precision payment methods for various tumors, alongside broadening health care reforms and optimizing policy measures, to guarantee the fair and sustainable distribution of medical resources. This approach aims to fulfill the patients’ demands for high-quality medical services. However, this study has several limitations, including the reliance on data and sample size from a single hospital and the lack of control for potential confounding factors. Future investigations need to expand the scope of samples, apply multivariable methods, and delve into the diversity of treatment protocols to enhance the generalizability and credibility of these findings.

## Data Availability

The data analyzed in this study is subject to the following licenses/restrictions: the data that support the findings of this study are available from the hospital quality control department but restrictions apply to the availability of these data, which were used under license for the current study, and so are not publicly available. Requests to access these datasets should be directed to the corresponding author. Requests to access these datasets should be directed to LX, xielj18@gmail.com.
